# Publisher Correction: A quantitative sequencing framework for absolute abundance measurements of mucosal and lumenal microbial communities

**DOI:** 10.1038/s41467-020-17055-1

**Published:** 2020-07-06

**Authors:** Jacob T. Barlow, Said R. Bogatyrev, Rustem F. Ismagilov

**Affiliations:** 1 Division of Biology and Biological Engineering, California Institute of Technology, 1200 East California Boulevard, Pasadena, CA USA; 2 Division of Chemistry and Chemical Engineering, California Institute of Technology, 1200 East California Boulevard, Pasadena, CA USA

**Keywords:** Biological techniques, Microbiology

Correction to: *Nature Communications* 10.1038/s41467-020-16224-6, published online 22 May 2020.

The original version of this Article contained an error in the Competing interests section. In the original article, it was incorrectly stated that the authors declare no competing interests; this statement omitted the following information: The authors declare the following competing interests: the technology described in this publication is the subject of a patent application filed by Caltech.

This has been corrected in both the PDF and HTML versions of the Article.

