# Supplementary material for: A quantitative sequencing framework for absolute abundance measurements of mucosal and lumenal microbial communities
Source: Nat Commun. 2020 May 22;11:2590. doi: 10.1038/s41467-020-16224-6 (PMC7244552; doi:10.1038/s41467-020-16224-6)
Supplement: Supplementary file 1 — Supplementary Information [file 41467_2020_16224_MOESM1_ESM.pdf]

Supplementary Info

A Quantitative Sequencing Framework for Absolute Abundance Measurements of Mucosal and Lumenal Microbial Communities

Jacob T. Barlow[1], Said R. Bogatyrev[1], Rustem F. Ismagilov[1,2]

[1]Division of Biology and Biological Engineering, California Institute of Technology
1200 E. California Blvd., Pasadena, CA, United States of America
[2]Division of Chemistry and Chemical Engineering, California Institute of Technology
1200 E. California Blvd., Pasadena, CA, United States of America
*Correspondence to: rustem.admin@caltech.edu

Supplementary Figures 1-9

Supplementary Tables 1-6

Supplementary References

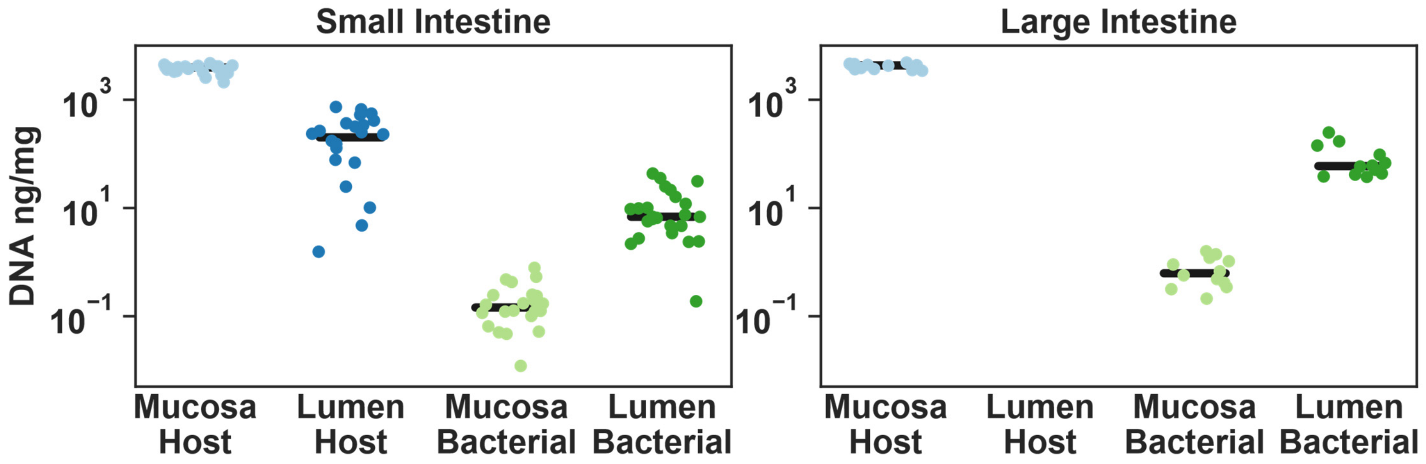

Supplementary Figure 1: Total DNA loads in small intestine and large intestine mucosa and lumen. Extracted DNA samples from mice in the ketogenic-diet group were measured by Nanodrop (total DNA) and digital PCR (microbial DNA). The horizontal lines represent the means and the points represent individual biological replicates (N = 24 for small intestine; N = 12 for large intestine).

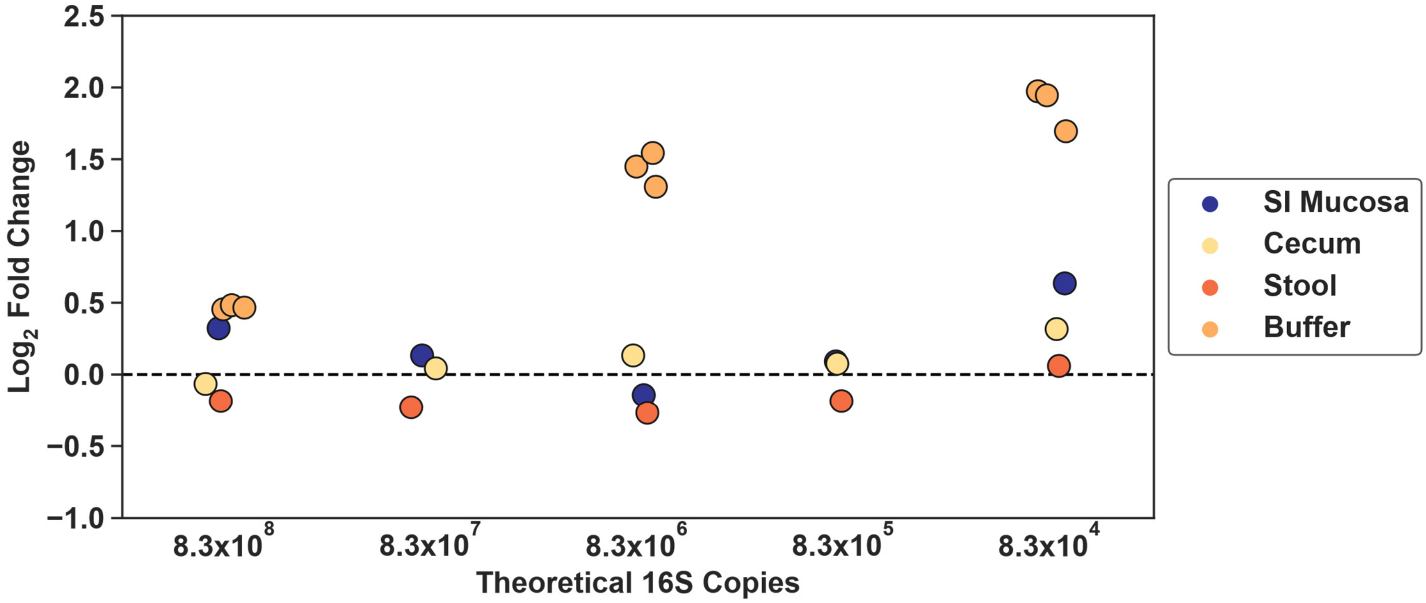

Supplementary Figure 2: Extraction and total DNA measurement accuracy of an eight-member mock microbial community dilutions spiked into extraction buffer or small-intestine mucosa, cecum, or stool from germ free mice. Log_2 fold change between theoretical and dPCR measured copies of 16S rRNA gene after extraction with varying input levels. Three technical replicates for buffer extractions are shown. All other sample types shown are N = 1 to illustrate the biological noise among sample types.

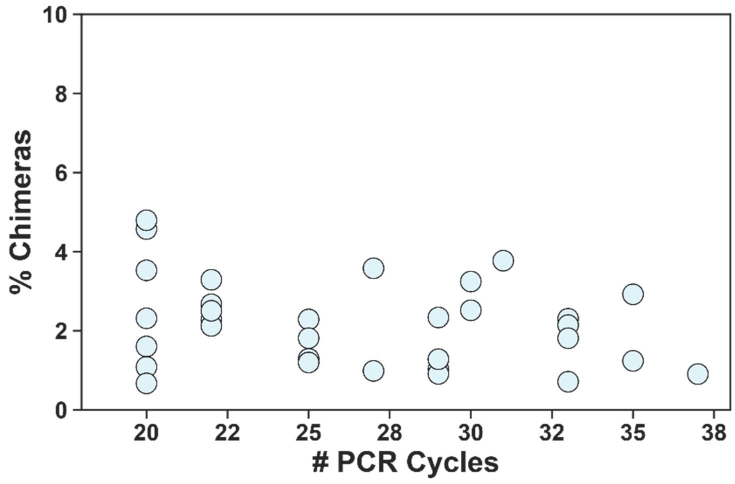

Supplementary Figure 3: Chimeric sequence prevalence is not determined by the number of PCR cycles. Relationship between the number of PCR cycles during the amplification reaction for library prep and the percentage of chimeric sequences detected by Divisive Amplicon Denoising Algorithm 2 (DADA2).[1] N = 33 samples that were sequenced from mice in the ketogenic-diet group.

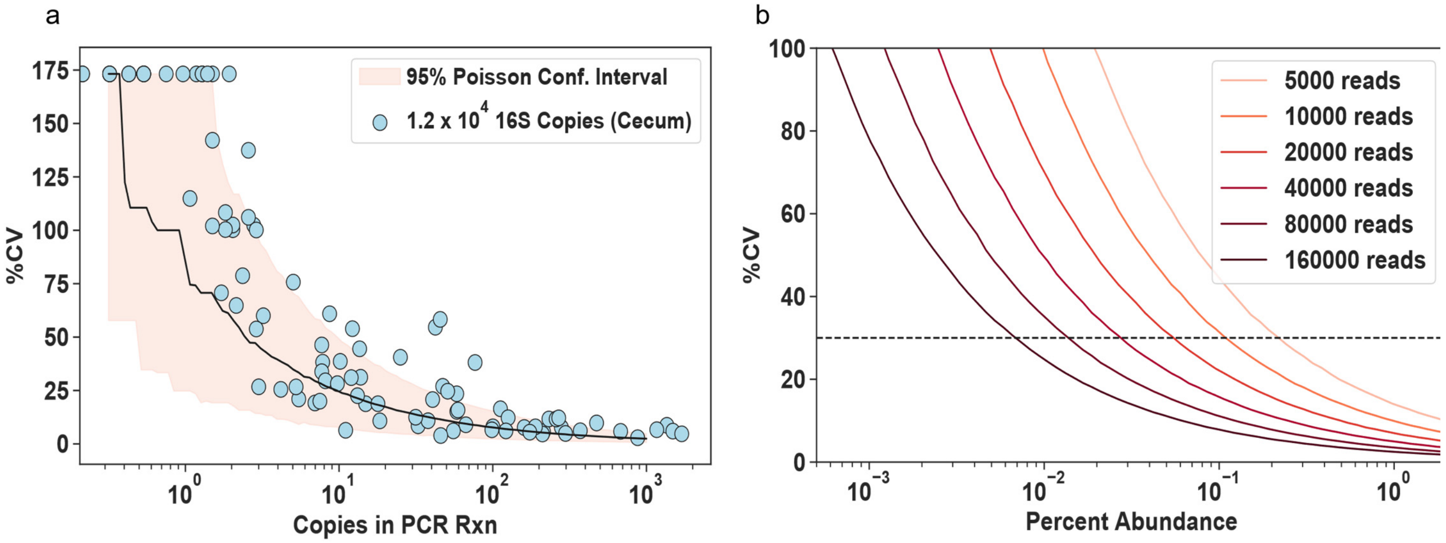

Supplementary Figure 4: Poisson limits of sequencing accuracy. (a) Relationship between the relative abundance of each taxon and % coefficient of variation (CV) using four technical (sequencing) replicates of a mouse cecum sample with an initial template input of 1.2 x 10^4 16S rRNA gene copies. The red shading indicates the bootstrapped (B = 10^4) Poisson sampling confidence interval of the input 16S rRNA gene copies. (b) Bootstrapped Poisson sampling relationship between %CV and percentage abundance as a function of read depth.

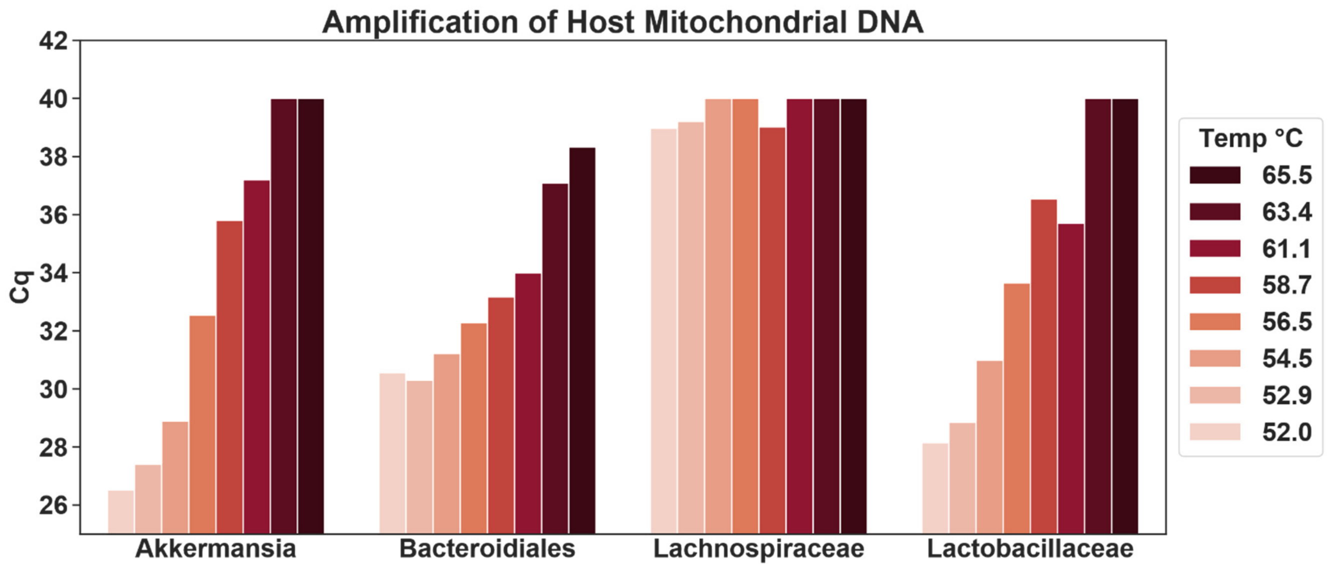

Supplementary Figure 5: Optimization of group-specific primers to eliminate amplification of host DNA. Relative abundance of non-specific product amplified from 20 ng/μL small-intestine mucosa sample from a germ-free mouse measured by qPCR. Lower Cq values indicate more amplification. Each color represents a different annealing temperature used during the cycling process. Samples were run in singlet at each temperature.

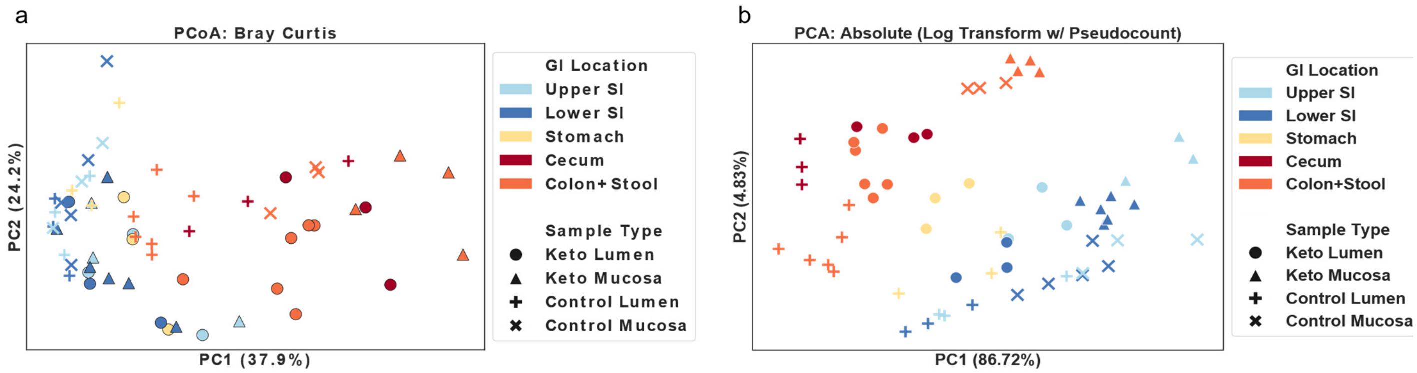

Supplementary Figure 6: Impact of ordination method on data visualization. (a) Principal coordinates analysis (PCoA) plot using Bray–Curtis dissimilarity metric of all samples collected 10 days after the diet switch. (b) Principal component analysis (PCA) plot using log-transform of absolute abundance data after adding a pseudocount of 1 read to all taxa.

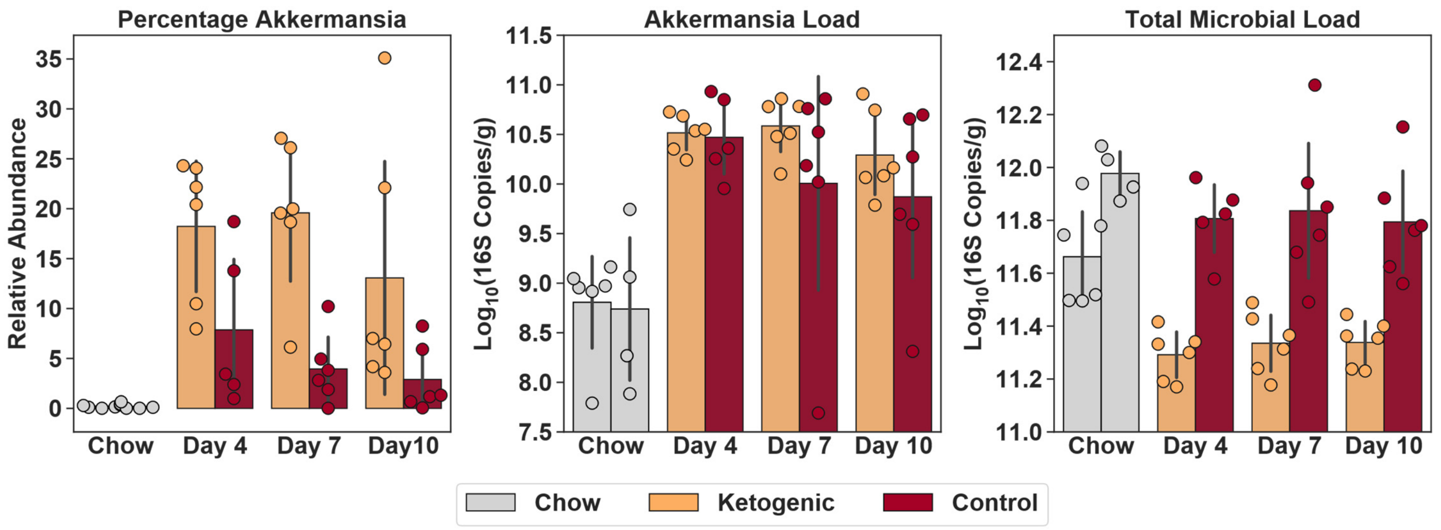

Supplementary Figure 7: Comparison of relative and absolute abundance quantification of *Akkermansia(g)* between mice on ketogenic and control diet. Average *Akkermansia(g)* load from stool of N = 6 mice on control diet (red) and N = 6 mice on ketogenic diet (orange). Grey points and bars indicate loads prior to the diet switch when all mice were on the chow diet. Data points from mice without *Akkermansia(g)* are not shown. Bar plots show mean plus or minus the standard deviation. Individual data points are overlaid on the bar plots.

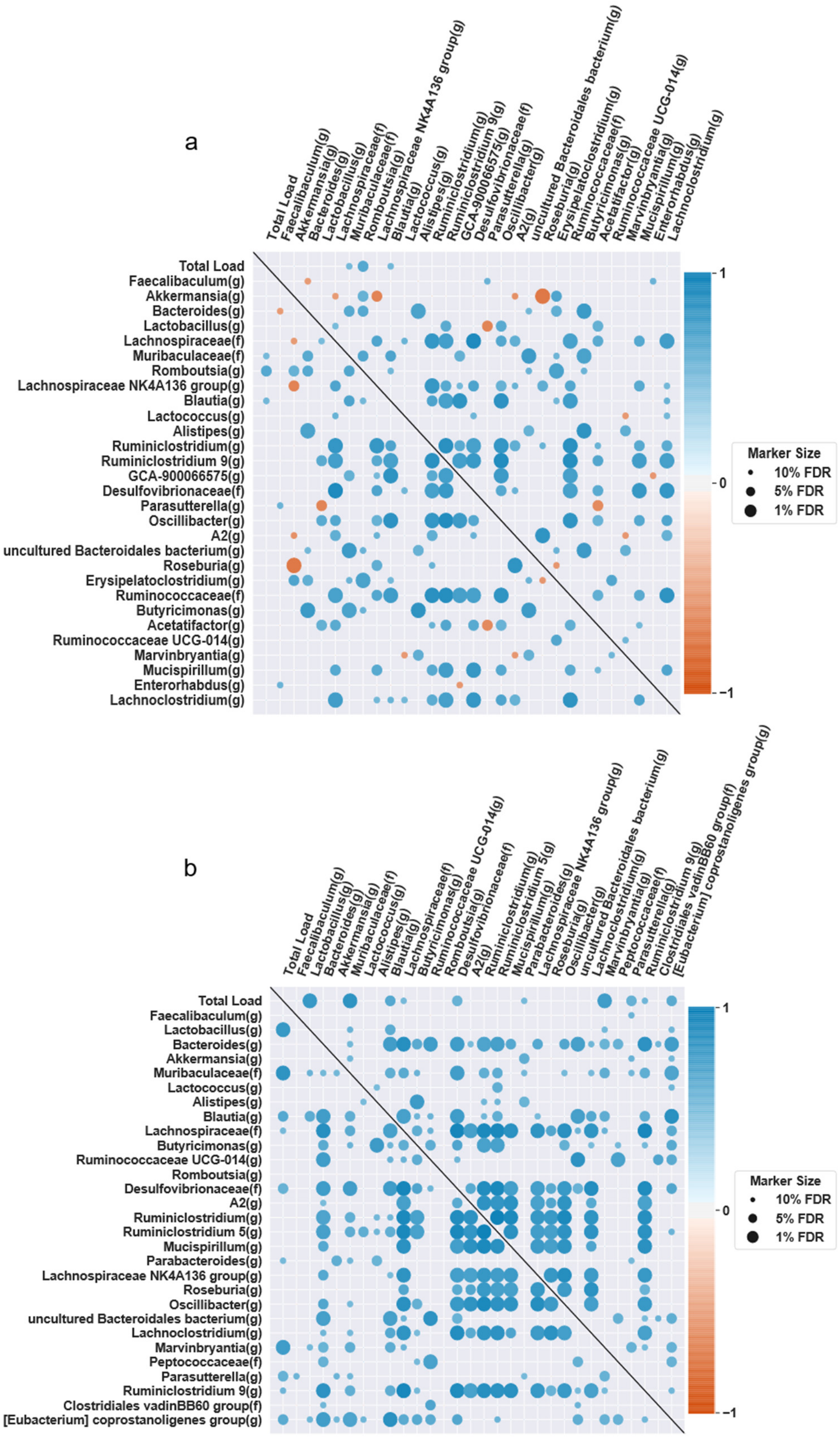

Supplementary Figure 8: Absolute-abundance measurements enable unbiased determination of correlation structure in microbiome datasets. Correlation matrices, using Spearman's rank, for the total microbial load and the top 30 most abundant taxa in stool samples from mice on either a ketogenic diet (a) or control diet (b). The color of each marker is based on the correlation coefficient (orange indicates negative correlations, blue indicates positive correlations) and the size is determined by the q-value of the correlation after Benjamini–Hochberg multiple testing correction. False-discovery rates (FDR) indicate the q-value at which the correlation was deemed significant: 1%, 5%, 10%. Abbreviations: (f), family; (g), genus; (o), order.

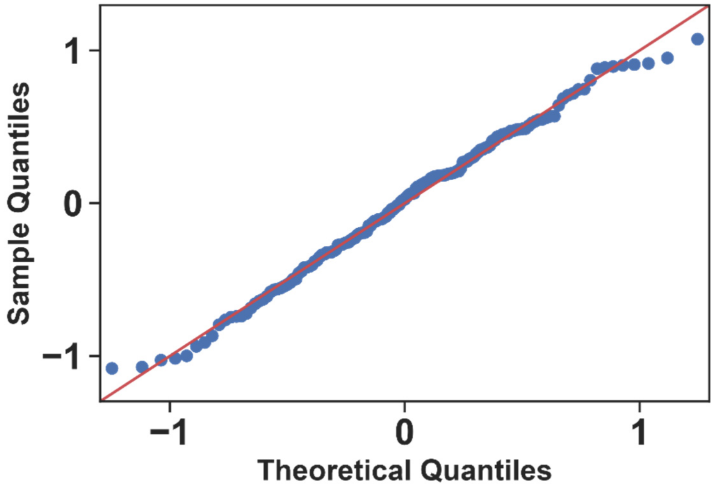

Supplementary Figure 9: The uncertainty in taxon absolute-abundance measures approximately follows a normal distribution. The quantile-quantile (Q–Q) plot of the mean-centered $\log_2$ relative error of absolute taxon abundances. The relative error is calculated as the ratio of the absolute taxon loads measured by our method of quantitative sequencing with dPCR anchoring over the absolute loads measured by taxon-specific primers in dPCR (data are from Fig. 3b). The x-axis represents the theoretical quantiles from a normal distribution while the y-axis is the actual quantiles of the mean-centered $\log_2$ relative errors.

Supplementary Table 1: Contaminant taxa with greater than 1% abundance in negative-control extraction.

Contaminant Taxa	Percentage Abundance
Acinetobacter(g)	31.38
Pseudomonas(g)	24.12
Allorhizobium-Neorhizobium-Pararhizobium-Rhizobium(g)	9.77
Brevundimonas(g)	5.86
Massilia(g)	2.84
Delftia(g)	2.52
Dietzia(g)	2.33
Corynebacterium 1(g)	2.08
Xanthomononadaceae(f)	2.06
Anaerococcus(g)	1.95
Nubsella(g)	1.94
Lysobacter(g)	1.91
Comamonas(g)	1.82
Janthinobacterium(g)	1.30
Shinella(g)	1.29
Novosphingobium(g)	1.23
Sphingobium(g)	1.15
Taibaiella(g)	1.02

(f), family; (g), genus

Supplementary Table 2: Comparison between digital PCR anchoring method for absolute abundance measurements and other published absolute abundance methods[2-5].

Approach	Major Improvement to the Field	Demonstrated Limit of Quantification	Demonstrated Limit of Detection	Demonstrated Precision	Validated Sampling Locations	Validation Against PCR Amplification Bias	Bias-Free Validation with High Host DNA Loads	References
**Flow Cytometry**	Showed importance of quantifying absolute abundance in clinical samples	Not Discussed	Not Discussed	Not Discussed	Stool	Not Applicable	Not Shown	Vandeputte *et al.* 2017[2]
**Sequencing Spike-ins**	Generated a variety of spike-in standards that can be used. Provided comprehensive analysis of detection limits and accuracy	Not Discussed	Dependent on spike-in amount (~100 copies/reaction)	1.5-1.7X with mock communities	Sludge, Soil	Show that it may skew total load measurement	Not Shown	Tourlousse *et al.* 2016[3]
**qPCR Anchoring**	Provided a simple and easy method for absolute quantification	Not Discussed	Not Discussed	High correlation at high DNA input levels	Stool	Not Discussed	Not Shown	Jian *et al.* 2018[4]
**Total DNA**	Provided a simple method for absolute quantification. Showed dramatic variability in loads across animal kingdom and clinical scenarios	Not Discussed	~100 pg of DNA	Not Discussed	Stool	Not Applicable	Not Applicable for stool	Contijoch *et al.* 2019[5]
**Digital PCR Anchoring**	Quantitative assessment of accuracy and precision of absolute abundances in complex gut samples and their impact on differential taxon analyses	4.2×10^5 16S copies/g Stool 1.0×10^7 16S copies/g Mucosa	4.2×10^4 16S copies/g Stool 1.0×10^6 16S copies/g Mucosa	2X across 6 orders of magnitude with low and high host DNA load	Stool, Mucosa, Small Intestine, Cecum, Stomach	Yes	Yes	This paper

Supplementary Table 3: Composition of ketogenic and control diets used in this study were based on previously reported diets (Envigo, Indianapolis, IN, USA).[6]

	TD.150300	TD.07797.PWD
	**Control Diet (g/kg)**	**Ketogenic Diet (g/kg)**
Casein	200	121
Crisco	61.25	605
Corn Oil	8.75	86.2
Cellulose	50	112.95
Corn Starch	389	0
Maltodextrin	100	0
Sucrose	150	0
DL-Methionine	3	1.56
Vitamin Mix, Teklad (40060)	10	17.8
Choline Bitartrate	0	2.5
TBHQ, antioxidant	0.07	0.14
Mineral Mix, Ca-P Deficient (79055)	13.37	23.8
Calcium Phosphate, dibasic	7.5	24.3
Calcium Carbonate	6.85	4.4
Magnesium Oxide	0.2	0.35

Supplementary Table 4: Absolute abundance, relative abundance, fold change and quantification class for each differentially abundant taxon in the stool 10 days after diet switch.

Taxon	Absolute Abundance Ketogenic Diet (16S copies/g)	Absolute Abundance Control Diet (16S copies/g)	log_2 Fold Change (Keto/Control)	Relative Abundance Ketogenic Diet (%)	Relative Abundance Control Diet (%)	Quantification Class
GCA-900066575(g)	1.94E+09	8.71E+07	4.00	0.799	0.014	Semi-Quant
Ruminococcaceae(f)	2.02E+09	2.43E+08	2.87	0.909	0.033	Semi-Quant
Lachnospiraceae NK4A136 group(g)	5.34E+09	8.57E+08	2.58	2.362	0.135	Quant
Acetatifactor(g)	5.20E+08	6.19E+07	2.46	0.256	0.009	Semi-Quant
Lachnospiraceae(f)	8.29E+09	1.71E+09	2.25	3.708	0.226	Quant
Ruminiclostridium 9(g)	1.91E+09	5.01E+08	1.84	0.863	0.059	Quant
Dorea(g)	7.79E+07	0.00E+00	1.36	0.032	0.000	Presence/Absence
Enterorhabdus(g)	7.55E+08	3.51E+08	0.99	0.349	0.052	Quant
[Eubacterium] xylanophilum group(g)	8.69E+07	1.91E+07	0.87	0.037	0.003	No Quant
Peptococcus(g)	8.83E+07	2.29E+07	0.79	0.040	0.004	Semi-Quant
Candidatus Soleaferrea(g)	5.91E+07	6.21E+06	0.78	0.026	0.002	No Quant
Marvinbryantia(g)	5.67E+08	1.35E+09	-1.26	0.226	0.218	Quant
Bacteroides(g)	8.81E+09	3.37E+10	-1.93	3.990	5.578	Quant
Faecalibaculum(g)	1.01E+11	3.87E+11	-1.93	46.724	54.268	Quant
Prevotellaceae UCG-001(g)	1.50E+07	7.87E+07	-2.12	0.006	0.015	No Quant
Bifidobacterium(g)	3.18E+08	1.42E+09	-2.15	0.153	0.100	Quant
Muribaculaceae(f)	1.25E+08	5.74E+08	-2.16	0.056	0.091	Quant
Ruminiclostridium 5(g)	2.85E+08	1.38E+09	-2.26	0.127	0.202	Quant
Ruminococcaceae UCG-014(g)	4.85E+08	2.45E+09	-2.32	0.209	0.427	Quant
Ruminococcaceae NK4A214 group(g)	8.66E+06	6.64E+07	-2.36	0.003	0.009	No Quant
Lactococcus(g)	3.34E+09	1.74E+10	-2.38	1.528	2.715	Quant
Muribaculaceae(f)	8.04E+09	4.26E+10	-2.40	3.520	6.506	Quant
Anaerotruncus(g)	2.23E+07	1.79E+08	-2.68	0.010	0.031	No Quant
Lactobacillus(g)	1.45E+10	1.35E+11	-3.22	6.632	19.295	Quant
Butyricimonas(g)	4.38E+08	4.39E+09	-3.30	0.203	0.755	Quant
Alistipes(g)	1.11E+09	1.15E+10	-3.36	0.526	2.018	Quant
Mollicutes RF39(o)	3.06E+07	3.99E+08	-3.38	0.014	0.074	Semi-Quant
Christensenellaceae(f)	2.35E+07	3.69E+08	-3.55	0.009	0.057	Semi-Quant
Clostridiales vadinBB60 group(f)	3.31E+07	5.68E+08	-3.77	0.017	0.108	Semi-Quant
ASF356(g)	0.00E+00	2.44E+08	-4.65	0.000	0.029	Presence/Absence
Parabacteroides(g)	9.26E+07	3.30E+09	-5.01	0.040	0.457	Quant
Gram-negative bacterium cTPY-13(g)	0.00E+00	3.71E+08	-5.20	0.000	0.050	Presence/Absence

(f), family; (g), genus; (o), order

Supplementary Table 5: Absolute abundance, relative abundance, fold change, and quantification class for each differentially abundant taxon in the lower small-intestine mucosa 10 days after diet switch.

Taxon	Absolute Abundance Ketogenic Diet (16S copies/g)	Absolute Abundance Control Diet (16S copies/g)	log_2 Fold Change (Keto/Control)	Relative Abundance Ketogenic Diet (%)	Relative Abundance Control Diet (%)	Quantification Class
Lachnoclostridium(g)	1.64E+07	7.07E+05	3.57	0.293	0.006	Semi-Quant
Lachnospiraceae(f)	9.60E+06	4.05E+05	3.16	0.171	0.006	Semi-Quant
A2(g)	2.29E+07	2.08E+06	3.06	0.441	0.025	Semi-Quant
Akkermansia(g)	2.57E+08	3.77E+07	2.74	5.576	0.419	Quant
Escherichia-Shigella(g)	2.96E+06	0.00E+00	2.21	0.059	0.000	Presence/Absence
Dorea(g)	2.94E+06	0.00E+00	2.20	0.062	0.000	Presence/Absence
Bacteroides(g)	5.51E+06	8.09E+05	1.94	0.112	0.009	Semi-Quant
Desulfovibrionaceae(f)	4.29E+06	5.91E+05	1.82	0.097	0.005	Semi-Quant
uncultured Bacteroidales bacterium(g)	1.55E+07	3.98E+06	1.76	0.365	0.041	Quant
Enterorhabdus(g)	1.12E+07	2.74E+06	1.73	0.245	0.022	Semi-Quant
Lachnospiraceae NK4A136 group(g)	1.38E+06	3.38E+05	0.65	0.031	0.005	No Quant
Ruminococcaceae(f)	8.48E+05	6.76E+04	0.51	0.017	0.001	No Quant
uncultured Lachnospiraceae bacterium(g)	6.46E+05	0.00E+00	0.35	0.013	0.000	Presence/Absence
Marvinbryantia(g)	4.62E+06	3.33E+06	0.27	0.127	0.037	Semi-Quant
Ruminiclostridium(g)	5.41E+05	0.00E+00	0.17	0.011	0.000	Presence/Absence
Muribaculaceae(f)	1.22E+08	3.48E+08	-1.51	2.585	3.041	Quant
Lactococcus(g)	1.20E+08	4.31E+08	-1.84	2.533	4.582	Quant
Lactobacillus(g)	9.05E+08	3.70E+09	-2.03	16.956	35.988	Quant

(f), family; (g), genus; (o), order

Supplementary Table 6: Primers used in this study, relevant conditions, and specificity. All primers were tested *in silico* for coverage of their desired taxonomic group and specificity.[7-13]

	Akkermansia muciniphila	**Bacteroidales**	***Lachnospiraceae***	***Lactobacillaceae***	**519F-806R**
Forward Primer	CAGCACGTGAAGGTGGGGAC	GGTGTCGGCTTAAGTGCCAT	CGGTACCTGACTAAGAAGC	GCAGCAGTAGGGAATCTTCCA	CAGCMGCCGCGGTAA
Reverse Primer	CCTTGCGGTTGGCTTCAGAT	CGGAYGTAAGGGCCGTGC	AGTTTYATTCTTGCGAACG	CACCGCTACACATGGAG	GGACTACHVGGGTWTCTAAT
Taxonomy Level	Species	Order	Family	Family	Kingdom
Annealing Temp (°C)	65	65	55	60	52
Concentration (nM)	500	500	500	500	500
Coverage (n=1 mismatch)	100%	75%	86%	91%	94% Bacteria, 95% Archaea
Potential Undetected Taxa	None	Rikenellaceae(f); Alistipes(g)	UCG-010(g)	None	None
Potential non-specific interactions (n=1 mismatch)	None	None	None	Leuconostocaceae(o)	None
Citation	Collado *et al*. (2007)[8]	Rinttilä *et al*. (2004)[9]	Kennedy *et al*. (2014)[10]	Castillo *et al*. (2006)[11]	Bogatyrev & Ismagilov (2020)[14]<br>Bogatyrev *et al*. (2020)[13]<br>Bogatyrev (2020)[12]

(f), family; (g), genus; (o), order

SUPPLEMENTARY REFERENCES

1. Callahan, B.J., McMurdie, P.J., Rosen, M.J., Han, A.W., Johnson, A.J.A. & Holmes, S.P. DADA2: High-resolution sample inference from Illumina amplicon data. *Nat. Methods* **13**, 581-583 (2016).
2. Vandeputte, D., Kathagen, G., D'hoe, K., Vieira-Silva, S., Valles-Colomer, M., Sabino, J., Wang, J., Tito, R.Y., De Commer, L., Darzi, Y., Vermeire, S., Falony, G. & Raes, J. Quantitative microbiome profiling links gut community variation to microbial load. *Nature* **551**, 507 (2017).
3. Tourlousse, D.M., Yoshiike, S., Ohashi, A., Matsukura, S., Noda, N. & Sekiguchi, Y. Synthetic spike-in standards for high-throughput 16S rRNA gene amplicon sequencing. *Nucleic Acids Res.* **45**, e23-e23 (2016).
4. Jian, C., Luukkonen, P., Yki-Järvinen, H., Salonen, A. & Korpela, K. Quantitative PCR provides a simple and accessible method for quantitative microbiome profiling. *PLoS One*, **15**, e0227285 (2020).
5. Contijoch, E.J., Britton, G.J., Yang, C., Mogno, I., Li, Z., Ng, R., et alw. Gut microbiota density influences host physiology and is shaped by host and microbial factors. *eLife* **8**, e40553 (2019).
6. Olson, C.A., Vuong, H.E., Yano, J.M., Liang, Q.Y., Nusbaum, D.J. & Hsiao, E.Y. The Gut Microbiota Mediates the Anti-Seizure Effects of the Ketogenic Diet. *Cell* **173**, 1728-1741.e1713 (2018).
7. Klindworth, A., Pruesse, E., Schweer, T., Peplies, J., Quast, C., Horn, M. & Glöckner, F.O. Evaluation of general 16S ribosomal RNA gene PCR primers for classical and next-generation sequencing-based diversity studies. *Nucleic Acids Res.* **41**, e1-e1 (2013).
8. Collado, M.C., Derrien, M., Isolauri, E., de Vos, W.M. & Salminen, S. Intestinal integrity and Akkermansia muciniphila, a mucin-degrading member of the intestinal microbiota present in infants, adults, and the elderly. *Appl. Environ. Microbiol.* **73**, 7767-7770 (2007).
9. Rinttilä, T., Kassinen, A., Malinen, E., Krogius, L. & Palva, A. Development of an extensive set of 16S rDNA-targeted primers for quantification of pathogenic and indigenous bacteria in faecal samples by real-time PCR. *J. Appl. Microbiol.* **97**, 1166-1177 (2004).
10. Kennedy, N.A., Walker, A.W., Berry, S.H., Duncan, S.H., Farquarson, F.M., Louis, P., Thomson, J.M., Satsangi, J., Flint, H.J., Parkhill, J., Lees, C.W. & Hold, G.L. The impact of different DNA extraction kits and laboratories upon the assessment of human gut microbiota composition by 16S rRNA gene sequencing. *PLoS One* **9**, e88982 (2014).
11. Castillo, M., Martín-Orúe, S.M., Manzanilla, E.G., Badiola, I., Martín, M. & Gasa, J. Quantification of total bacteria, enterobacteria and lactobacilli populations in pig digesta by real-time PCR. *Vet. Microbiol.* **114**, 165-170 (2006).
12. Bogatyrev, S.R. Development of Analytical Tools and Animal Models for Studies of Small-Intestine Dysbiosis. *Dissertation (Ph.D.), California Institute of Technology*, doi:10.7907/VJDZ-7B52 (2020).
13. Bogatyrev, S.R., Rolando, J.C. & Ismagilov, R.F. Self-reinoculation with fecal flora changes microbiota density and composition leading to an altered bile-acid profile in the mouse small intestine. *Microbiome*, **8**. doi: 10.1186/s40168-020-0785-4 (2020).
14. Bogatyrev, S.R. & Ismagilov, R.F. Quantitative microbiome profiling in lumenal and tissue samples with broad coverage and dynamic range via a single-step 16S rRNA gene DNA copy quantification and amplicon barcoding. *Preprint available at:* https://biorxiv.org/cgi/content/short/2020.01.22.914705v1 (2020).
