# Supplementary material for: A quantitative sequencing framework for absolute abundance measurements of mucosal and lumenal microbial communities
Source: Nat Commun. 2020 May 22;11:2590. doi: 10.1038/s41467-020-16224-6 (PMC7244552; doi:10.1038/s41467-020-16224-6)
Supplement: Supplementary file 2 — Reporting Summary [file 41467_2020_16224_MOESM2_ESM.pdf]

nature research

Corresponding author(s): Rustem Ismagilov

Last updated by author(s): April 1, 2020

Reporting Summary

Nature Research wishes to improve the reproducibility of the work that we publish. This form provides structure for consistency and transparency in reporting. For further information on Nature Research policies, see Authors & Referees and the Editorial Policy Checklist .

Statistics

For all statistical analyses, confirm that the following items are present in the figure legend, table legend, main text, or Methods section.

n/a	Confirmed	
☐	☒	The exact sample size (*n*) for each experimental group/condition, given as a discrete number and unit of measurement
☐	☒	A statement on whether measurements were taken from distinct samples or whether the same sample was measured repeatedly
☐	☒	The statistical test(s) used AND whether they are one- or two-sided<br>*Only common tests should be described solely by name; describe more complex techniques in the Methods section.*
☐	☒	A description of all covariates tested
☐	☒	A description of any assumptions or corrections, such as tests of normality and adjustment for multiple comparisons
☐	☒	A full description of the statistical parameters including central tendency (e.g. means) or other basic estimates (e.g. regression coefficient) AND variation (e.g. standard deviation) or associated estimates of uncertainty (e.g. confidence intervals)
☐	☒	For null hypothesis testing, the test statistic (e.g. *F*, *t*, *r*) with confidence intervals, effect sizes, degrees of freedom and *P* value noted<br>*Give P values as exact values whenever suitable.*
☒	☐	For Bayesian analysis, information on the choice of priors and Markov chain Monte Carlo settings
☒	☐	For hierarchical and complex designs, identification of the appropriate level for tests and full reporting of outcomes
☒	☐	Estimates of effect sizes (e.g. Cohen's *d*, Pearson's *r*), indicating how they were calculated

Our web collection on statistics for biologists contains articles on many of the points above.

Software and code

Policy information about availability of computer code

Data collection	Quantasoft analysis pro V1.7.4.0917 was used for collection of all digital PCR measurements.
Data analysis	Processing of all sequencing data was performed using QIIME 2 2019.1. Raw sequence data were demultiplexed and quality filtered using the q2-demux plugin followed by denoising with DADA2. Beta-diversity metrics were estimated using the q2-diversity plugin. Taxonomy was assigned to amplicon sequence variants (ASVs) using the q2-feature-classifier classify-sklearn naive Bayes taxonomy classifier against the Silva61 132 99% OTUs references from the 515F/806R region. All downstream analyses were performed in Python v3.7 using IPython v7.13.0 with the following packages: NumPy v1.18.1, Pandas v1.0.3, seaborn v0.10.0.rc0, matplotlib v3.1.3, scipy v1.4.1, sklearn v0.22.1

For manuscripts utilizing custom algorithms or software that are central to the research but not yet described in published literature, software must be made available to editors/reviewers. We strongly encourage code deposition in a community repository (e.g. GitHub). See the Nature Research guidelines for submitting code & software for further information.

Data

Policy information about availability of data

All manuscripts must include a data availability statement. This statement should provide the following information, where applicable:

- Accession codes, unique identifiers, or web links for publicly available datasets
- A list of figures that have associated raw data
- A description of any restrictions on data availability

The complete sequencing data generated during this study are available in the National Center for Biotechnology Information Sequence Read Archive repository under study accession number PRJNA575097. Raw data for all figures available through CaltechDATA: https://data.caltech.edu/records/1371. Data underlaying all figures is also provided as Source Data files.

Field-specific reporting

Please select the one below that is the best fit for your research. If you are not sure, read the appropriate sections before making your selection.

☒ Life sciences ☐ Behavioural & social sciences ☐ Ecological, evolutionary & environmental sciences

For a reference copy of the document with all sections, see nature.com/documents/nr-reporting-summary-flat.pdf

Life sciences study design

All studies must disclose on these points even when the disclosure is negative.

Sample size	No sample size calculation was performed. Cohort sizes were determined based on previous findings (Olsen et al., Cell 2018).
Data exclusions	One of the mice never carried Akkermansia and thus was excluded from calculations based on Akkermansia. Several stool samples during longitudinal collection were unable to be collected and thus data is missing. When determining differential taxa only genera present in at least 66% of mice on one of the diets were included.
Replication	Variation in technical replications of all measurements were shown to be smaller than the presented variation in bioligical replications. Each experiment was performed once with replicates as indicated. Repeat experiments were not performed.
Randomization	Mice were randomly assigned to study groups and placed in cages by matching weight (n=2 mice per cage).
Blinding	Investigators were not blinded during data collection and analyses. This was due to the fact that animal experiments were meant as a model to illustrate the advantages of our absolute abundance methods over standard relative abundance methods instead of a way to glean the effects of the ketogenic diet on mice.

Reporting for specific materials, systems and methods

We require information from authors about some types of materials, experimental systems and methods used in many studies. Here, indicate whether each material, system or method listed is relevant to your study. If you are not sure if a list item applies to your research, read the appropriate section before selecting a response.

Materials & experimental systems

n/a	Involved in the study	
☒	☐	Antibodies
☒	☐	Eukaryotic cell lines
☒	☐	Palaeontology
☐	☒	Animals and other organisms
☒	☐	Human research participants
☒	☐	Clinical data

Methods

n/a	Involved in the study	
☒	☐	ChIP-seq
☒	☐	Flow cytometry
☒	☐	MRI-based neuroimaging

Animals and other organisms

Policy information about studies involving animals; ARRIVE guidelines recommended for reporting animal research

Laboratory animals	4-week old, female, Swiss Wester mice and 2-6 month old male and female Germ-Free C57Bl/6J mice were used in this study. Mice were housed on heat-treated hardwood chip bedding (Aspen Chip Bedding, Northeastern Products, Warrensburg, NY, USA) and provided with tissue paper (Kleenex, Kimberly-Clark, Irving, TX, USA) nesting material. Experimental animals were fed standard chow (Lab Diet 5010), 6:1 ketogenic diet (Harlan Teklad TD.07797; Table S3) or vitamin- and mineral-matched control diet (Harlan Teklad TD.150300; Table S3). To minimize cage effects, mice were housed two per cage with three cages per diet group. Custom feeders, tin containers approximately 2.5 inches tall with a 1-inch diameter hole in the top, were used for the ketogenic diet as it is a paste at room temperature. Autoclaved water was provided ad lib and cages were subjected to a daily 13:11 light:dark cycle throughout the study.
Wild animals	This study did not involve wild animals
Field-collected samples	This study did not involve samples collected from the field
Ethics oversight	All animal husbandry and experiments were approved by the Caltech Institutional Animal Care and Use Committee (IACUC protocols #1646 and #1769)

Note that full information on the approval of the study protocol must also be provided in the manuscript.
